# Ability of Nafamostat Mesilate to Prolong Filter Patency during Continuous Renal Replacement Therapy in Patients at High Risk of Bleeding: A Randomized Controlled Study

**DOI:** 10.1371/journal.pone.0108737

**Published:** 2014-10-10

**Authors:** Yong Kyu Lee, Hae Won Lee, Kyu Hun Choi, Beom Seok Kim

**Affiliations:** 1 Nephrology Division, Internal Medicine Department, National Health Institute Corporation, Ilsan Hospital, Goyang, Republic of Korea; 2 Nephrology Division, Department of Internal Medicine, Severance Hospital, Yonsei University College of Medicine, Seoul, Republic of Korea; University Medical Center Utrecht, Netherlands

## Abstract

Continuous renal replacement therapy (CRRT) is considered as an effective modality for renal replacement therapy in hemodynamically unstable patients within intensive care units (ICUs). However, the role of heparin anticoagulation, which is used to maintain circuit patency, is equivocal due to the risk of bleeding and morbidity. Among various alternative anticoagulants, nafamostat mesilate has been shown to be an effective anticoagulant in patients prone to bleeding. Hence, we conducted a prospective, randomized controlled study investigating the effect of nafamostat mesilate on mortality, CRRT filter life span and adverse events in patients with bleeding tendency. Seventy-three Patients were randomized into either the futhan or no-anticoagulation group. Thirty-six subjects in the futhan group received nafamostat mesilate, while thirty seven subjects in the no-anticoagulation group received no anticoagulants. Baseline characteristics and appropriate laboratory tests were taken from each group. The mortality between the two groups was not significantly different. Nevertheless, between the futhan group and the no-anticoagulation group, the overall number of filters used during CRRT (2.71±2.12 vs. 4.50±3.25; *p = 0.042*) and the number of filters changed due to clots per 24 hours (1.15±0.81 vs. 1.74±1.62; *p = 0.040*) were significantly different. When filter life span was subdivided into below and over 12 hours, the number of filters functioning over 12 hours was significantly higher in the futhan group than in the no-anticoagulation group (p = 0.037, odds ratio 1.84). There were no significant differences in transfusion, mortality, or survival between the two groups, and no adverse events related to nafamostat mesilate were noted. Hence, nafamostat mesilate may be used as an effective and safe anticoagulant, without increasing the risk of major bleeding complications, in patients prone to bleeding.

**Trial Registration:**

Clinicaltrials.gov NCT01761994

## Introduction

Continuous renal replacement therapy (CRRT) is an effective renal replacement modality used to manage hemodynamically unstable patients with deteriorated renal function [1]. In CRRT, anticoagulants are generally used to prevent circuit coagulation, and heparin is used most commonly, in this regard. However, there are risks associated with the use of heparin as an anticoagulant in patients at high risk of bleeding. Thus, modified anticoagulation methods, such as low dose heparin, low molecular weight heparin, regional citrate, regional unfractionated heparin, thrombin antagonists, and prostacyclin anticoagulation, are used to ensure filter patency and patient safety in these patients despite their limitations and adverse events [2]–[7]. Nafomostat mesilate (6-amno-2-naphthyl p-guanidinobenzoate dimethane sulfonate; Futhan, SK chemicals, Seoul, Republic of Korea) is a prostacyclin analog that inhibits serine proteases and is rapidly eliminated from blood with a half-life of 8 minutes. The extremely short half-life makes it a suitable substitute for heparin in patients with a high tendency for bleeding [8]–[10]. Even though a few retrospective studies have shown that nafamostat mesilate is effective in CRRT among patients at high risk of bleeding [11]–[13], no prospective study has evaluated the effect of nafamostat mesilate under controlled conditions. Accordingly, to elucidate the efficacy and safety of nafamostat mesilate, we performed a single center, randomized, controlled study in CRRT patients with high risk of bleeding.

## Methods

The protocol for this trial and supporting CONSORT checklist are available as supporting information; see Checklist S1 and Protocol S1.

### Patients and study design

In this unblinded, single center, randomized, prospective controlled study, 73 patients (18–80 years old) who were admitted to the intensive care unit (ICU) for CRRT with hemorrhagic tendency were enrolled from September 2007 to August 2010 at Severance Hospital, Seoul, Republic of Korea. Patients were included if they required CRRT and had at least one of the following hemorrhagic tendencies: (1) platelet count <100,000/µL, (2) activated partial thromboplastin time>60 seconds, (3) prothrombin time-international normalized ratio>2.0, (4) active hemorrhage, (5) surgery within the past 48 hours, (6) cerebral hemorrhage within the past 3 months or history of a major cerebral bleeding, and (7) septic shock or disseminated intravascular coagulation. Patients who were pregnant (or possibly pregnant), breast feeding, allergic to nafamostat mesilate, or had any other conditions that made the candidate unfit according to the attending physician were excluded. The patients were followed for 1 to 23 days until CRRT discontinuation. The Institutional Review Board of Severance Hospital approved this study, and all patients or their legal representative provided written informed consent. Since the registration of the trial to a recognized international registry was not mandatory during Institutional Review Board approval, the registration was completed during the study. The authors confirm that all ongoing and related trials for this drug are registered.

### Randomization and Treatment allocation

At enrollment, the patients were assigned randomly with stratification of diabetes mellitus. The patients who fulfilled the inclusion criteria and none of the exclusion criteria were assigned to either the futhan group or no-anticoagulation group according to the random assignment number by preformed random place card.

### Outcomes

The primary outcome of this study was to assess the mortality of the futhan group and compare it to the mortality of the no-anticoagulation group. The secondary outcome was to evaluate filter life span (overall filter, filter containing clot at exchange, filter changed due to clotting), transfusion, and adverse events.

### Covariates

Vital signs including pulse rate and blood pressure were checked when CRRT was initiated. Data on patient demographics and underlying diseases were collected at screening. In addition, laboratory examinations including hematologic, biochemical, and coagulation tests were done at screening and at the cessation of CRRT. If patients died during CRRT, the last examination before death was used. Overall mortality, mortality during hospitalization, and mortality 28 days after CRRT were compared between groups to evaluate the safety of nafamostat mesilate.

Filter life span using the filter patency time and the reason for filter failure (e.g., filter clot, ultrafiltrate loss <150 mL/hr within 3 hours, persistent transmembrane pressure higher than 200 mmHg, or an extracorporeal circuit abnormality due to another apparatus [such as radiologic examination], etc.) were evaluated. When CRRT was discontinued, the average life span of the filter was calculated. If the last filter was discontinued due to death or the discretion of the clinician, it was excluded from analysis to derive a more exact life span of CRRT filters.

### CRRT Setting

Central venous access was achieved by placing a double lumen catheter into the internal jugular or femoral veins. CRRT was conducted using Prisma (Gambro, Lund, Sweden) or Prismaflex (Gambro). A commercially prepared bicarbonate-buffered replacement fluid (Hemosol B0, Gambro) was used as a dialysate and replacement fluid. Blood flow was set between 130 mL/min and 200 mL/min, and ultrafiltration rates were at least 35 mL/(hr·kg). Replacement fluid was delivered by the predilution mode. Filters were electively exchanged every 48 hours, if they were not discontinued due to malfunction of the filter due to various reasons, death, or at the request of the physician.

The initial dose of nafamostat mesilate was 20 mg/hr. The dosage was adjusted from 10 mg/hr to 30 mg/hr according to each patient's status. For priming, two vials of nafamostat mesilate were dissolved in 2 mL of 5% glucose fluid and mixed with 1000 mL of normal saline. After carefully removing air bubbles from the circuit with the prepared fluid, nafamostat mesilate was dissolved with 15 mL of 5% glucose fluid and loaded into the anticoagulation line with a starting dose of 20 mg/hr. The nafamostat mesilate was administered throughout the CRRT duration in futhan group.

In the no-anticoagulation group, no placebo medication was administered.

### Transfusion

Packed red blood cells were transfused when hemoglobin level decreased below 7 g/dL or below 10 g/dL with evidence of acute bleeding. Platelet concentrates are transfused when the platelet level decreased below 20,000/µL or 50,000/µL with evidence of acute bleeding. Fresh frozen plasma was transfused when prothrombin time fell below 70% with evidence of bleeding or if disseminated intravascular coagulation was suspected.

### Adverse events

Physical examination was performed to collect data on allergies and cardiovascular, pulmonary, gastrointestinal, hepatobiliary, endocrinologic, nephrologic, urologic, muscular, neurologic, and psychiatric backgrounds at the screening before beginning the CRRT and after CRRT by the same researcher. Adverse events were categorized using the World Health Organization Adverse Reaction Terminology (WHO-ART) [14]. The severity of the adverse events was categorized using The Common Terminology Criteria for Adverse Events (CTCAE) [15]. Relations between adverse events and medications were categorized as (1) definitely related, (2) probably related, (3) possibly related, (4) probably not related, and (5) definitely not related.

### Statistics

All variables were analyzed using SPSS for Windows version 18.0 (SPSS, Inc., Chicago, IL, USA). Data are expressed as means ± standard deviation. Comparisons between the futhan group and no-anticoagulation group were conducted using Student's t-tests. Chi-square tests were used to compare frequency measurements between the two groups. Logistic regression analyses were used to compare the statistical significance of each category within adverse events. Kaplan-Meyer estimator was used in survival curve. The comparison between survival curves were performed by log-rank test. All *p*-value less than 0.05 were considered statistically significant.

Sample size calculation was performed using GPower version 3.01. Considering previous study, we hypothesized that the rate of primary outcome would be 50% in patients undergoing CRRT [1], [4], [5]. A priori power calculations estimated that a minimum of 31 subjects in each arm would enable us to detect 15% allowable error in mortality (alpha  = 0.01). Considering a 5% drop-out rate during the study, 31 subjects were determined to be sufficient.

## Results

### Baseline characteristics

**Figure 1:**
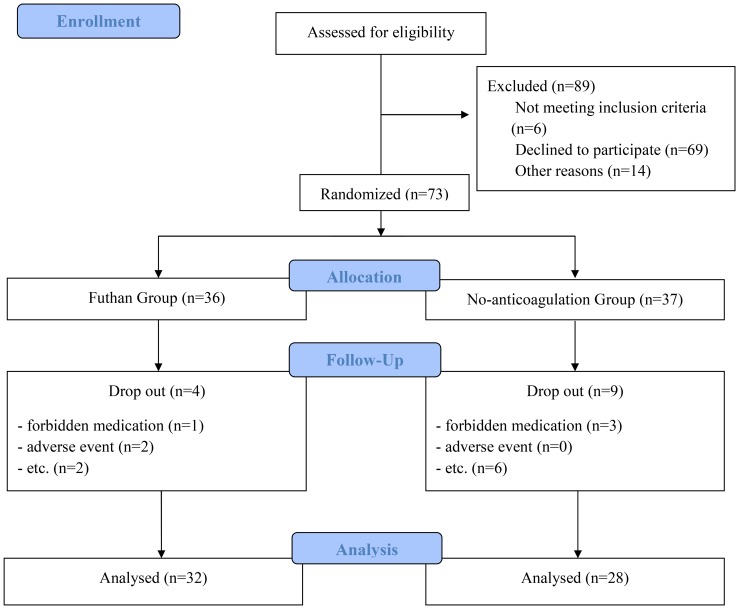
Enrollment, randomization, and follow up. Out of 162 patients who were eligible to the study, 73 patients were enrolled in the study, and 60 patients completed the study for analysis.

Out of the 73 enrolled patients, 60 patients completed the study; four patients from the futhan group, and nine patients from the no-anticoagulation group were dropped out. Reasons for drop out are shown in Figure 1, such as prescribing drugs that would compromise the study, adverse events, etc. Thus, the final number of patients in the futhan group and the no-anticoagulation group were 32 and 28, respectively. At the start of CRRT, no significant differences between groups according to age, sex, vital signs, laboratory tests, or acute kidney injury when stratified by RIFLE criteria, APACHE II score, and the Cleveland Clinical Foundation Score were found (Table 1). There was no significant difference between groups in laboratory test at the cessation of CRRT (Table not included).

**Table 1:** Baseline characteristics.

Characteristics	Futhan group	No-anticoagulation group	*P value*
	(n = 36)	(n = 37)	
Demographics			
Age (years)	52.97±13.94	57.54±13.04	*0.152*
Male, N (%)	24 (66.67%)	20 (54.05%)	*0.271*
Underlying disease, N(%)			
Hypertension	14 (38.9%)	13 (36.1%)	*0.808*
Diabetes mellitus	13 (36.1%)	8 (22.2%)	*0.195*
Vital signs			
SBP (mmHg)	122.42±20.89	121.03±21.33	*0.779*
DBP (mmHg)	66.75±15.39	63.68±12.44	*0.350*
Pulse rate (bpm)	113.36±24.27	113.35±23.10	*0.999*
Body temperature (°C)	36.68±0.81	36.81±1.14	*0.585*
RR (/min)	19.71±4.58	20.05±4.62	*0.755*
Laboratory tests at start of CRRT			
WBC (×10^3^/ 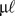 )	12.45±11.11	10.49±9.88	*0.427*
Hb (g/dL)	8.49±1.55	9.07±1.86	*0.147*
Platelet (×10^3^/ 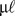 )	57.44±40.05	90.92±97.39	*0.087*
ESR (mm/hr)	22.70±25.34	26.67±34.52	*0.920*
Uric acid (mg/dL)	7.47±2.93	7.05±2.58	*0.224*
BUN (mg/dL)	64.09±25.64	61.71±30.16	*0.385*
Cr (mg/dL)	3.09±1.09	3.41±1.96	*0.718*
Na (mmol/L)	140.28±8.00	140.81±7.49	*0.774*
K (mmol/L)	4.19±0.82	4.24±1.06	*0.843*
Total CO_2_ (mmol/L)	20.63±6.21	21.22±4.96	*0.288*
Patient severity index at screening.			
RIFLE criteria			
Risk	4 (11.1%)	9 (24.30%)	*0.140*
Injury	10 (27.8%)	8 (21.6%)	*0.542*
Failure	22 (61.1%)	18 (51.3%)	*0.285*
Loss and ESRD	0	1 (2.7%)	*0.493*
Total APACHE II score	26.72±5.26	26.84±6.00	*0.931*
Cleveland clinical foundation score	17.31±11.11	13.73±3.25	*0.071*

### Mortality

**Figure 2:**
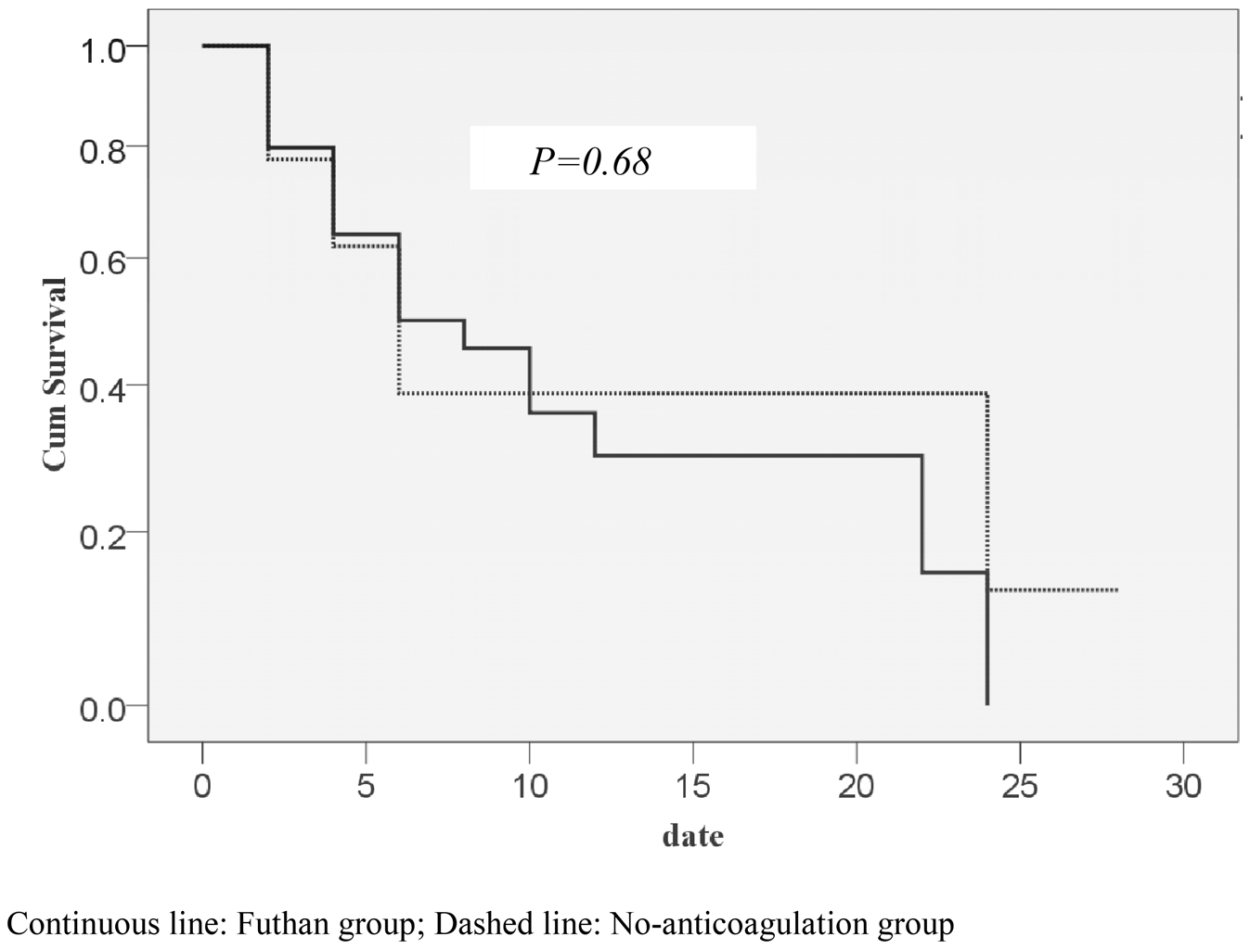
Survival curve of the Futhan group and No-anticoagulation group.

Although the overall mortality was higher than expected, both groups showed similar overall mortality (futhan: 75.00%, n = 24 vs. no-anticoagulation: 74.07%, n = 20; *p = 0.927*). When patients were stratified by prevalence of diabetes mellitus or their APACHE II score, no significant difference between the groups was found; however, diabetic patients showed higher mortality than that in non-diabetic patients. Mortality during hospitalization was similar between the groups (futhan: 71.88%, n = 23 vs. no-anticoagulation: 74.07%, n = 20; *p = 0.963*). Also, mortality on 28 days after applying CRRT was not significantly different between the two groups (futhan: 75.00%, n = 24 vs. no-anticoagulation: 74.07%, n = 20; *p = 0.927*) (Table 2). Median survival in the futhan group and no-anticoagulation group was 3.96 and 4.42 days, respectively (*p = 0.680*) (Figure 2). There were no significant differences in median survival between the two groups, when we stratified overall mortality according to prevalence of diabetes, RIFLE criteria, and APACHE II scores (data not shown).

**Table 2:** Comparison of mortality in each group.

Mortality	Futhan group	No-anticoagulation group	*P value*
Overall mortality	24 (75.00%)	20 (74.07%)	*0.927*
Mortality on 28 days	23 (71.88%)	20 (74.07%)	*0.963*
Mortality within hospital	24 (75.00%)	20 (74.07%)	*0.927*

### Filter life span

The only significant change between the futhan and no-anticoagulation group was found in the overall number of filters changed during CRRT and the number of filters changed due to clots per 24 hours. Filter life span tended to be longer in the futhan group than in the no-anticoagulation group, although without statistical significance. Also, the number of filters used during CRRT tended to be higher in the no-anticoagulation group than the futhan group, without statistical significance (Table 3). Interestingly, when filter life span was subdivided into below and over 12 hours, the number of filters functioning over 12 hours was significantly higher in the futhan group than the no-anticoagulation group. Hence, we can assume that filters are likely to be functional for a longer time in the futhan group than in the no-anticoagulation group (Table 4).

**Table 3:** Distribution of filter life spans in each group.

	Futhan group	No-anticoagulation group	Total
≤12 hrs	57 (41.3%)	26 (27.7%)	83 (35.8%)
>12 hrs	81 (58.7%)	68 (72.3%)	62 (64.2%)
Total	138	94	232

P = 0.037; odd ratio 1.840.

**Table 4:** Comparison of filters consumed in each group.

	Futhan group	No-anticoagulation group	*P value*
Filter life span (hours)			
Overall filters	26.63±21.14	22.70±20.67	*0.160*
Filters with clots	26.03±20.27	21.25±19.49	*0.106*
Filters changed due to clots	27.05±20.29	23.23±19.61	*0.221*
Number of filters used in the ICU			
Overall filters	2.71±2.12	4.50±3.25	*0.042*
Filters with clots	86.2%	77.7%	*0.111*
Filters changed due to clots	73.4%	72.5%	*1.000*
Number of filters/24 hours			
Overall filters	1.60±1.67	1.90±1.60	*0.383*
Filters with clots	1.45±1.57	1.85±1.62	*0.378*
Filters changed due to clots	1.15±0.81	1.74±1.62	*0.040*

### Transfusion

The number of platelet concentrate transfusions was significantly lower in the futhan group than the no-anticoagulation group. However, there was no significant difference in the number of packed red blood cells and fresh frozen plasma transfusions between the two groups (Table 5).

**Table 5:** Comparison of transfusion in each group.

Transfusion (packs)	Futhan group	No-anticoagulation group	*P value*
RBC	3.19±3.18	3.56±3.79	*0.22*
Platelet concentrate	24.31±25.64	38.67±50.16	*<0.01*
FFP	6.72±8.06	6.72±8.06	*0.28*

### Adverse events

There were 52 adverse events from 33 patients in the futhan group and 59 events from 33 patients in the no-anticoagulation group (*p = 0.133*). In the futhan group, there were 4 cardiologic events, 11 pulmonary events, 9 gastrointestinal events, 2 hematologic events, 2 nephrologic events, 1 gynecologic event, 3 neurologic events, 2 dermatologic events, and 20 infectious events. There were five adverse events which were related to bleeding in the futhan group. The bleeding consisted of one grade 1 pulmonary hemorrhage, one grade 4 gastrointestinal bleeding, two grade 2 gastrointestinal bleedings, and one grade 1 vaginal bleeding. However, there were no adverse events related to nafamostat mesilate. Pulmonary hemorrhage resulted from cardiopulmonary resuscitation, and vaginal bleeding was due to dysfunctional uterine bleeding which was resolved with medroxyprogesterone. Out of three gastrointestinal bleedings, one severe incidence was due to thrombocytopenia by allopurinol, while the other two incidences were due to ulcer and resolved by transfusion and medication. Out of 59 adverse events in the no-anticoagulation group, there were 7 cardiologic events, 9 pulmonary events, 2 hepato-biliary events, 8 gastrointestinal events, 7 hematologic events, 2 endocrinologic events, 1 gynecologic event, 1 neurologic event, 2 dermatologic events, and 20 infectious events. There were also five adverse events that were related to bleeding in the no-anticoagulation group. The adverse events included a variceal bleeding, two grade 2 events of gastrointestinal bleeding, one grade 1 gastrointestinal bleeding, and one catheter insertion site oozing. Logistic regression analysis of the frequency of each adverse event showed no statistical difference between the two groups.

## Discussion

Nafamostat mesilate is a synthetic serine protease inhibitor originally developed as a therapy for pancreatitis. However, due to its inhibitory function on platelet aggregation and coagulation factors, such as thrombin, Xa, XIIa, kallikrein, and complement system components, nafamostat mesilate has been used more commonly since 1990 (mainly in Japan) as an anticoagulant in CRRT. There are no absolute contraindications in using nafamostat mesilate as an anticoagulant in patients who are planning to receive CRRT. This is a strong advantage for nafamostat mesilate, compared to the characteristic side effects and contraindications of other anticoagulants. However, nafamostat mesilate is not accepted as a standard anticoagulant for CRRT due to limited evidence [16]–[18].

In this study, we evaluated the effect of nafamostat mesilate as an anticoagulant with a randomized, prospective, controlled study protocol. The mortality rate in our study was significantly higher compared to others with similar APACHE II score. This is probably due to the fact that the subjects enrolled in our study comprised a bleeding tendency with needs for CRRT, which would add more severity, when compared to other subjects with the same APACHE II score. Nevertheless, overall mortality, mortality during hospitalization, mortality at 28 days, and median survival were not statistically different between the two groups, despite concerns for severe bleeding in the futhan group. Although the overall number of filters changed within 24 hours was not significantly different, comparison of the number of filters changed due to clotting per 24 hours showed that the futhan group required significantly fewer filters than the no-anticoagulation group did. Also, when the groups were subdivided according to filter life span over and below 12 hours, significantly more filters were maintained over 12 hours in the futhan group than the no-anticoagulation group. CRRT is a labor intensive procedure requiring constant attention by health care providers, and our results suggest that nafamostat mesilate can reduce the workload of health care providers, cost, and eventually improve patient outcomes by reducing the time spent preparing CRRT due to recurrent filter failure.

Currently, regional citrate anticoagulation is recommended in the 2012 Kidney Disease Improving Global Outcome (KDIGO) Clinical Practice Guidelines for Acute kidney Injury for patients with bleeding tendencies [19]. This conclusion was drawn from several clinical studies showing the advantages of citrate in comparison to heparin in terms of prolonged filter life span, reduced hemorrhagic incidence, and lower transfusion requirement [20]–[25]. However, since citrate is metabolized by the liver, citrate is applied cautiously in patients with severe liver failure, septic or cardiogenic shock, and impaired citrate metabolism. But, the patients who are planned to undergo CRRT, will most likely have decreased liver or cardiac function, sepsis, or conditions that can lead to impaired citrate metabolism. As a result, there is the possibility for acid-base imbalance, electrolyte abnormalities, hypotension and arrhythmia, which can be life-threatening by itself, but would not be a concern in nafamostat mesilate [26], [27]. Since it is recommended to change CRRT filters in at least every 72 hours, filters were changed every 48 hours in the present study, even though the filter was not clotted. Hence, overall filter life span in the present study was shorter than other studies performed with citrate, in which the median filter life span was about 120 hours. If we were to use citrate in our clinical setting, the filter life span would not be as long as 120 hours and probably would be similar to the filter life span that we observed in the futhan group. Hence, further studies are required to compare the clinical advantages between citrate and nafamostat mesilate.

The transfusion of packed red blood cells and fresh frozen plasma during CRRT was not significantly different between the two groups. This result indicates that bleeding risk due to nafamostat mesilate may be negligible. However, a significantly smaller amount of platelet concentrates transfusion was required in the futhan group, and this might have resulted from higher platelet consumption in the no-anticoagulation group, which was due to filter clotting.

There have been several reports of circuit clotting and adverse events, including anaphylaxis, agranulocytosis, and hyperkalemia with nafamostat mesilate [28]–[31]. Hence, we investigated adverse events in the present study. Two patients dropped out from the study in the futhan group due to adverse events that led to discontinuation of nafamostat mesilate. The events were severe hyperbilirubinemia (grade 3) and moderately elevated prothrombin time (grade 2) that were “definitely” not related to treatment with nafamostat mesilate. There were no bleeding adverse events related to nafamostat mesilate. The 52 adverse events observed in the futhan group during the study were also “definitely” not related to nafamostat mesilate, except for one incidence of gastrointestinal bleeding that was “probably” not related to the medication. The comparison of the frequency of each adverse event indicated that nafamostat mesilate is as safe as no-anticoagulation treatment.

### Limitations

The limitation of this study was a higher drop-out rate than expected. A priori power analysis showed 31 subjects in each arm to be sufficient to detect a meaningful difference in mortality. Considering 5% drop-out rate, 33 patients in each arm were considered sufficient for the study. However, during the study, a large number of patients than expected dropped out in the no-anticoagulation group, despite the fact that we have enrolled 36 subjects in the futhan group and 37 subjects in the no-anticoagulation group. The final number of patients in the no-anticoagulation group was 28, while that in the futhan group was 32. However, although the number of subject was insufficient, there have been statistically significant advantages in filter patency. Hence, further study, preferably a multi-centered study, might reveal more noticeable advantages for using nafamostat mesilate in CRRT patients with bleeding tendencies.

### Conclusions

This prospective, randomized, controlled study confirmed that nafamostat mesilate prolongs filter life span without any added adverse events. These results suggest that nafamostat mesilate is a safe and effective anticoagulant in CRRT patients at high risk of bleeding.

## Supporting Information

Checklist S1 **CONSORT checklist.**
(DOC)

Protocol S1 **Trial protocol.**
(PDF)
