# Supplementary material for: Ability of Nafamostat Mesilate to Prolong Filter Patency during Continuous Renal Replacement Therapy in Patients at High Risk of Bleeding: A Randomized Controlled Study
Source: PLoS One. 2014 Oct 10;9(10):e108737. doi: 10.1371/journal.pone.0108737 (PMC4193755; doi:10.1371/journal.pone.0108737)
Supplement: Protocol S1 — Trial protocol. (PDF) [file pone.0108737.s002.pdf]

SK chemical.co.Ltd

Study Protocol

| | |
|---|---|
| Study No: | FTN_401 |
| Protocol Version: | version 1.00 |
| Study Drug: | Futhan (nafamostat mesilate) |
| Study Title: | Ability of nafamostat mesilate to prolong filter patency during continuous renal replacement therapy in patients with a high risk of bleeding |
| Study Phase: | Phase 4 |
| P.I.: | Beom Seok Kim, MD. PhD. Department of Internal Medicine, Severance Hospital, Seoul, Korea |
| Institution: | Severance Hospital, Seoul, Korea |
| Funding: | SK chemicals corp. |
| Duration: | 12 months from protocol approval |
| Confidential | © SK chemicals corp.<br>This protocol is the property of SK chemicals corp. and may not – in full or in part – be passed on, reproduced, published or otherwise use without the express permission of SK chemicals corp. |

Index


**■ Abbreviation and terminology** **7**

**■ Study Protocol Summary** **8**

**■ Study schedule** **11**

**1. Clinical study title and phase** **13**

1.1 Title 13

1.2 Phase 13

**2. Institution, principal investigator/pharmacist and funding** **14**

2.1 Clinical study institution 14

2.2 Principal investigator and co-investigator 14

2.2.1 Principal investigator 14

2.2.2 Co-investigator 14

2.3 Clinical study pharmacist 14

2.4 Client 14

2.5 Clinical research organization 14

**3. Purpose & Background** **15**

3.1 Purpose 15

3.1.1 Primary end point 15

3.1.2 Secondary end point 15

3.2 Background 15

**4. Medications in clinical study** **18**

4.1 Overview of study medication 18

4.1.1 Study medication 18

4.2 Production and packing of medication used in clinical study 20

4.3 Maintenance and revocation of double blind status 20

**5. Target disease and clinical study period** **21**

5.1 Target disease 21

5.2 Clinical study period 21
**6. Inclusion/exclusion criteria, target number of subjects and its estimation basis 22**
6.1 Inclusion criteria 22
6.2 Exclusion Criteria 22
6.3 Target number of subjects and its estimation basis 22
6.3.1 Number of subjects 22
6.3.2 Estimation basis 23
**7. Clinical study method 24**
7.1 Clinical study design 24
7.2 Dosage, administration method and administration duration 24
7.2.1 Dosage 24
7.2.2 Administration method 24
7.2.3 Administration duration 25
7.3 CRRT method 25
7.4 Combination criteria 25
7.4.1 Possible combination medication 25
7.4.2 Medication that are prohibited to use in combination 25
7.5 Randomization 26
**8. Observation, clinical examination and observation method 27**
8.1 Clinical study schedule 27
8.2 Observation 28
8.2.1 Written consent and demographical survey 28
8.2.2 Past history and drug history survey 28
8.2.3 Vital sign 29
8.2.4 Physical examination 29
8.2.5 Pregnancy test and Clinicopathologic examination 29
8.2.6 Central venous pressure monitoring 30
8.2.7 Severity evaluation 30
8.2.8 Filter life span 31
8.2.9 Subject suitability assessment 31
8.2.10 Randomization and prescription of study medication 31

8.2.11 Identification of change in medication history 31
8.2.12 Collection of medication and checking compliance 32
8.2.13 Checking adverse events 32
8.2.14 Checking renal function 32
8.2.15 Checking Mortality 32
8.2.16 Blood loss 33
8.2.17 Transfusion 33
8.3 Observation method (per visit) 33
8.3.1 Screening 33
8.3.2 Medication phase (randomization and after initiation of CRRT) 33
8.3.3 Study completion or discontinuation 34
8.3.4 Follow-up 34
**9. Precautions using study medication** **36**
9.1 Precaution considering the study medication: SK chemicals corp. ‘IV futhan’ 36
**10. Study discontinuation, drop out, or analysis exclusion criteria** **39**
10.1 Study discontinuation and drop out criteria 39
10.2 Disposal of violation on protocol and non-compliance 39
**11.Efficacy assessment criteria, assessment method, and analysis method (Data analysis method)** **41**
11.1 Assessment criteria 41
11.1.1 Primary validity assessment variable 41
11.1.2 Secondary validity assessment variables 41
11.2 Assessment method 41
11.2.1 Primary validity assessment variable 41
11.2.2 Secondary validity assessment variables 41
11.3 Analysis method 43
11.3.1 General principals of result analysis 43
11.3.2 Demographic baseline data 43
11.3.3 Validity data 44
11.3.3.1 Primary validity assessment value 44
11.3.3.2 Secondary validity assessment value 44
11.3.4 Safety data 45

11.3.4.1 Adverse event 45
11.3.4.2 Clinicopathologic examination result 45
11.3.4.3 Bleeding accident 45
**12. Safety evaluation method including adverse event, evaluation criteria and reporting method 46**
12.1 Definition of safety related terms 46
12.2 Evaluation method 47
12.3 Evaluation criteria 47
12.3.1 Fever 47
12.3.2 Subjective/objective sign 47
12.4 Cause-and-effect relationship with the study medication 48
12.5 Reporting method 49
12.5.1 Reporting the adverse event 49
12.5.2 Prompt report 50
12.6 Measures taken when serious adverse drug reaction occurs 52
**13. Consent form, compensation agreement, and patient's treatment after clinical study 54**
13.1 Consent form 54
13.2 Agreement on compensation 54
13.3 Criteria for patient's treatment after clinical study 54
**14. Measures on patient's safety 55**
14.1 Clinical study institution 55
14.2 Approval and modification of the study protocol 55
14.3 Thorough awareness of the study protocol 55
14.4 Consent to clinical study 55
14.5 Accurate selection of subjects 55
14.6 Inspection of clinical study progression 56
14.7 Monitoring of clinical study institution 56
14.8 Subject's confidentiality 56
14.9 Management of clinical study medication 57
14.10 Management after adverse event 57
**15. Other provision that is necessary to perform the clinical study safely and scientifically 58**

| | |
|---|---|
| **16. Reference** | **59** |
| **Enclosure 1. Signature page** | **60** |
| **Enclosure 2. Agreement on patient's compensation** | **61** |
| **Enclosure 3. Manual and consent form for patient** | **62** |
| **Enclosure 4. Declaration of Helsinki** | **71** |
| **Enclosure 5. Criteria for adverse event** | **79** |
| **Enclosure 6. Criteria for DIC** | **80** |
| **Enclosure 7. APACHE II score** | **81** |
| **Enclosure 8. CCF score** | **85** |
| **Enclosure 9. RIFLE score** | **86** |

■ Abbreviation and terminology

| | |
|---|---|
| Study Medication | : Futhan (Nafamostat mesilate) |
| ADR | : Adverse Drug reaction |
| AE | : Adverse event |
| ALT | : Alkaline transaminase |
| ALP | : Alkaline phosphatase |
| APTT | : Activated partial thromboplastin time |
| ARDS | : Adult respiratory distress syndrome |
| AST | : Aspartate transaminase |
| AT | : Angiotensin |
| BUN | : Blood urea nitrogen |
| CCT | : Celite coagulation time |
| CRF | : Case Report Form |
| CRO | : Contract Research Organization |
| CRRT | : Continuous renal replacement therapy |
| CVVHDF | : Continuous venovenous hemodiafiltration |
| DIC | : Diffuse intravascular coagulation |
| ECG | : Electro cardiography |
| ESR | : Erythrocyte Sedimentation Rate |
| FUT | : Futhan |
| Hb | : Hemoglobin |
| Hct | : Hematocrit |
| Hep | : Heparin |
| HUS | : Hemolytic uremic syndrome |
| ICU | : Intensive care unit |
| INR | : International Normalized Ratio |
| IRB | : Institutional Review Board |
| ITT | : Intention-To-Treat |
| KFDA | : Korea Food & Drug Administration |
| No | : Number |
| PP | : Per Protocol |
| SD, std | : Standard Deviation |
| SGOT | : Serum generation oxaloacetic transaminase |
| SGPT | : serum generation pyruvic transaminase |
| SOP | : Standard Operation procedure |
| WHO | : World Health Organization |

■ Study Protocol Summary

| | |
|---|---|
| Title | Ability of nafamostat mesilate to prolong filter patency during continuous renal replacement therapy in patients with a high risk of bleeding |
| Funding | SK chemicals corp. |
| Institution & P.I. | Beom Seok Kim MD. PhD.<br>Department of Internal medicine, Severance Hospital, Seoul, Korea |
| Study Duration | Patients were enrolled 12 months after IRB approval |
| Subjects | Patients who are in need of CRRT in ICU setting with a bleeding tendency that prohibits use of heparin. |
| End Point | · Primary End Point<br>Mortality in ICU<br>· Secondary End Point<br>Comparison of efficacy and safety in two method during CRRT by comparing in-hospital mortality, mortality after 28 days after CRRT, CRRT duration, ICU period, in-hospital period, survival Time, filter life span, Blood loss, Transfusion, etc. |
| Phase & Design | Phase IV<br>Randomized, parallel phase IV clinical study |
| Study Drug | Futhan (nafamostat mesilate) |
| Dosage & Administration Method | IV futhan 50mg 1 vial was dissolved with 15 mL of 5% glucose solution. Futhan was initiated with a dose of 20 mg/hr, which was adjusted between 10 to 30 mg/hr under strict supervision of researchers. For priming, 2 vials of futhan was dissolved in 2mL of 5% glucose solution and injected to 500mL normal saline bag. This fluid was used to<br>IV futhan |
| Administration Duration | Until cessation of CRRT |

■ Study Protocol Summary

<table>
<tr><td>No of subjects</td><td>(1) Level of significance, ($\alpha = 0.1$)<br>(2) Allowable limits of error for mortality are 15%. ($\delta$=0.15)<br>(3) Considering the patient's severity in ICU, wastage rate is set at 5%. ($r$ = 0.05)
<table>
<tr><td></td><td>Futhan group</td><td>No-anticoagulation group</td><td>Total</td></tr>
<tr><td>Validity assessment subject</td><td>31</td><td>31</td><td>62</td></tr>
<tr><td>Validity assessment subject including drop-out (5%)</td><td>33</td><td>33</td><td>66</td></tr>
</table></td></tr>
<tr><td>Inclusion Criteria</td><td>(1) Male/female adult patients aged from 18 to 80<br>(2) Patients who are hemodynamically unstable and in need of CRRT with one of following hemorrhagic tendencies:<br>① Platelet count < 100,000<br>② aPTT > 60sec<br>③ PT-INR > 2.0<br>④ Active bleeding<br>⑤ Major surgery within 48 hours<br>⑥ History of cerebral hemorrhage or major bleeding within 3 months<br>⑦ Septic shock or DIC<br>(3) Patients or his/her legal guardian has voluntarily agreed to participate in the study and provided written informed consent.</td></tr>
<tr><td>Exclusion Criteria</td><td>(1) Pregnancy, breast feeding or possibility of pregnancy<br>(2) History of allergy to nafamostat mesilate that might lead to shock.<br>(3) Any other conditions that made the candidate unfit according to the attending physician.</td></tr>
</table>

■ Study Protocol Summary

| | |
|---|---|
| Method | This study is a single-center, randomized, open clinical study to evaluate mortality, patients' recovery rate, safety between nafamostat mesilate group and no-anticoagulation group in patients who are prohibited from using heparin.<br><br>When the ICU patient who has hemorrhagic tendency and in need of CRRT voluntarily agrees to participate in the clinical study, the patients who are suitable by inclusion criteria are randomly assigned to each group. Nafamostat mesilate will be administered until CRRT is ceased due to various reasons, and after which patients mortality within 28 days after CRRT initiation and renal functional recovery at 1st outpatient department follow up. |
| Validity assessment | · primary end point: mortality within ICU<br>· secondary end point<br>① In-hospital mortality<br>② Mortality within 28 days after initiation of CRRT<br>③ CRRT duration<br>④ ICU hospitalization period<br>⑤ Total hospitalization period<br>⑥ Survival<br>⑦ Filter life span<br>⑧ Blood loss<br>⑨ transfusion |
| Safety assessment | Adverse events, clinical pathology examination (hematologic, blood coagulation test, lipid exam, urine analysis) |

■ Study schedule

All clinical study should follow planned schedule.

| Period | Screening | Treatment[1] | Closing[2] | Follow-up[7] |
|---|---|---|---|---|
| Written consent | X | | | |
| Authorize a Screening number | X | | | |
| Demographical analysis | X | | | |
| Personal/Drug history survey | X | | | |
| Vital sign check[3] | X | X | X | |
| Physical examination | X | | X | |
| Pregnancy test[4] | X | | | |
| Clinicopathologic examination[5] | X | X[5] | X | |
| Severity analysis | X | | | |
| Filter life span | | X | X | |
| CVP monitoring | | X | | |
| Subject compatibility assessment[6] | | X | | |
| Randomization | | X | | |
| Drug administration | | X | | |
| Check difference in drug dosage | | X | X | |
| Adverse event check | | X | X | |
| Residual renal function check[8] | | | | X |
| Mortality check | | | X | X |
| Blood loss | | X | X | |
| Packed RBC transfusion | | X | X | |
| Free frozen plasma, Platelet[9] | | X | | |

1: administered until treatment is concluded

2: If patients are improved, closing examination was performed at discontinuation of CRRT. However, if the patient has deceased, the last examination before death was used.

3: Body temperature, pulse rate, respiratory rate, blood pressure was examined before other examination was performed. During CRRT, vital sign was examined every 4 hours. At screening, body temperature was examined rectally.

4: Pregnancy test was performed in female without sterilization or any effective contraception in child-bearing age.

5: Detailed examination was as following;

· Hematologic: Hemoglobin, Hematocrit, RBC, WBC, Platelet, differential count, ESR

· Biochemical: Alkaline phosphatase, BUN, Creatinine, SGPT (ALT), SGOT (AST), Total bilirubin, Uric acid, Na, K, Cl, Ca, P, CRP, total $CO_2$

· Arterial Blood Gas: $O_2$, $CO_2$, arterial pH, $HCO_3$, Base excess/deficit, Oxygen saturation

· Lipid: Cholesterol

· Urine analysis: pH, Protein, Glucose, Ketone, WBC, RBC

· Coagulation test: aPTT, PT-INR

· Specific coagulation test: fibrinogen, fibrin degradation product

During CRRT, Clinicopathologic examination was performed every 72 hours. If CRRT was discontinued before 72 hours, examination was performed at discontinuation. Uine analysis was performed at screening and closing. Specific coagulation examination was substituted for the examination performed recently

6: Subject compatibility assessment was performed based on all examination, personal history and inclusion/exclusion criteria. Patients who were fit in these criteria was enrolled as subjects.

7: The patients were followed until discharge for mortality to confirm survival until 28 days after initiation of CRRT.

8: After discontinuation of CRRT, patients were followed at discharge to confirm dialysis maintenance and at first outpatient department follow-up after discharge, renal functional restoration was checked.

9: Free frozen plasma, platelet transfusion was checked during CRRT.

# 1. Clinical study tile and phase

## 1.1 Title

Ability of nafamostat mesilate to prolong filter patency during continuous renal replacement therapy in patients with a high risk of bleeding

## 1.2 Phase

Phase IV

## 2. Institution, principal investigator/pharmacist and funding

### 2.1 Clinical study institution

Severance hospital, 134, Shinchondong, Seodaemungu, Seoul, Korea

### 2.2 Principal investigator and co-investigator

#### 2.2.1 Principal investigator

Beom Seok Kim, MD. PhD. Department of internal medicine, Severance hospital

#### 2.2.2 Co-investigator

Jeong Eun Lee, MD. Department of internal medicine, Severance hospital
Seong Jin Mun, MD. Department of internal medicine, Severance hospital

### 2.3 Clinical study pharmacist

Severance hospital

### 2.4 Funding

SK chemicals. Corp
948-1, Daechi3dong, Kangnamgu, Seoul, Korea

### 2.5 Clinical research organization

Dream CIS
Jeoksun Hyundail building 10-10, 80, Jeoksundong, Jongrogu, Seoul, Korea

## 3. Purpose & Background

Randomized, open, phase IV clinical study to compare mortality, patient's recovery after CRRT, etc. between nafamostat mesilate and no-anticoagulation during CRRT in patients who are not fit to use heparin in ICU setting.

### 3.1 Purpose

#### 3.1.1 Primary end point

We are planning to compare mortality in ICU between nafamostat mesilate group and no-anticoagulation group in patients who are not fit to use heparin due to hemorrhagic tendency.

#### 3.1.2 Secondary end point

We compared CRRT duration, ICU, hospitalization duration, survival, filter life span, blood loss, and transfusion between two groups and efficiency and safety of nafamostat mesilate.

### 3.2 Background

Continuous renal replacement therapy is a modality that dialyzes for 24 hours a day to minimize physical burden. This method is superior in hemodynamical stability, correction of metabolic acidosis and nutritional deficiency to conventional hemodialysis due to its biocompatibility [1].

Generally to maximize efficiency of dialysis, heparin is applied to prevent coagulation in extracorporeal circuit. However, it is difficult to use anticoagulation such as heparin in patients with hemorrhagic tendency. In these cases, regional anticoagulation with protamine reversal, low molecular weight heparin, regional citrate anticoagulation, prostacyclin anticoagulation, and no-coagulation is applied as alternative method, but these methods have complications and limitations [2].

The phase III clinical study performed in Japan which compared nafamostat mesilate and heparin in hemodialysis patient with hemorrhagic tendency showed that nafamostat mesilate group had smaller difference in hemorrhagic tendency compared to heparin group and there were no significant difference in residual blood in extracorporeal circuit in both group. The hemostasis time after completion of hemodialysis was significantly shorter in nafamostat mesilate group compared to heparin group and change in arterial blood coagulation test was also significantly shorter in nafamostat mesilate group compared to heparin group at 1 hour after initiation of dialysis.

The phase III study about effect of nafamostat mesilate on bleeding and coagulation in hemodialysis patients with hemorrhagic tendency shows that there hemorrhage was not increased in any of the patients, 71% of patients showed decreased hemorrhage, 29% of patients showed no difference in nafamostat mesilate group, while 4% showed increased hemorrhage, 68% of patients with no difference in hemorrhage, 28% of patients showed decreased hemorrhage in low molecular weight heparin group. Residual blood in the circuit was significantly fewer in nafamostat mesilate group and coagulation in drip-chamber at venous side was lowered in nafamostat mesilate group [3].

Heparin usage during hemodialysis in patients with hemorrhagic complication or after operation may aggravate hemorrhage. Nafamostat mesilate which is a strong protease inhibitor has a short half life, thus the anticoagulation effect is limited to the extracorporeal circuit. The phase III study in Japan and Korea showed that nafamostat mesilate is a safe anticoagulation to be used in hemodialysis patients with hemorrhagic tendency.

In clinical setting, patients who are on CRRT tend to have multi-organ failure with sepsis. And CRRT can reduce renal burden and remove septic mediator in septic shock patients with acute renal failure [1]. Since, DIC occurs due to intravascular coagulation abnormality due to various reasons, which leads to microemboli in multiple organs and results in tissue necrosis, organ failure and hemorrhagic tendency by consumption of intravascular coagulation factors.

When dialysis is performed without heparin, there are several limitations that high volume of normal saline is required and blood flow is needed to be maintained high. Hence efficient dialysis is hard to achieve.

In this study, we will compare mortality and patients' recovery after CRRT between patients with nafamostat as anticoagulation and patients with no-anticoagulation.

4. Medications in clinical study

4.1 Overview of study medication

4.1.1 Study medication

· Name: Futhan IV
· Active principal: nafamostat mesilate
· Dose: nafamostat mesilate 10 or 50mg/vial
· Character: white powder in clear vial
· Storage: at room temperature (1~30℃)
· Volume of active principal
10mg of nafamostat mesilate in IV futhan
50mg of nafamostat mesilate in IV futhan 50
· Effect:

① Relieve acute symptom of pancreatitis (acute pancreatitis, acute phase of chronic pancreatitis, post-Op acute pancreatitis, acute pancreatitis after pancreatic duct imaging, traumatic pancreatitis)

② Disseminated intravascular coagulation

③ Anticoagulation of extracorporeal circuit on patients with hemorrhagic disease or tendency

· Dosage

⑴ Administration

① To relieve acute symptom of pancreatitis

: Generally, 10mg of nafamostat mesilate is dissolved in 500mL of 5% glucose fluid and administered intravenously for 2 hours once or twice daily. Decrease or increase on symptom.

② DIC

: 0.06~0.20 mg/Kg of nafamostat mesilate is dissolved in 1,000mL of 5% glucose fluid and administered intravenously for 24hours continuously.

③ To anticoagulate the extracorporeal circuit on patients with hemorrhagic disease or tendency

: For priming, two vials of nafamostat mesilate were dissolved in 2 mL 5% glucose fluid and mixed with 1000 mL normal saline. After carefully removing air bubbles from the circuit with the prepared fluid, nafamostat mesilate was dissolved with 15 mL of 5% glucose fluid and loaded into the anticoagulation line with a starting dose of 20~50 mg/hr. In clinical study, average dose of maintenance was 35mg/hour.

⑵ Making IV fluids

To properly administer nafamostat mesilate, IV fluid should be prepared in following order.

① To relieve acute symptom of pancreatitis

ⓐ Add more than 1mL of 5% glucose fluid in 10mg nafamostat mesilate vial and dissolved fully.

ⓑ Mix fully dissolved fluid into 500mL of 5% glucose fluid.

② DIC

ⓐ Add more than 1mL of 5% glucose fluid in 10mg vial and more than 5mL of 5% glucose fluid in 50mL vial until fully dissolved.

ⓑ Mix fully dissolved fluid into 1,000mL of 5% glucose fluid.

③ To anticoagulate the extracorporeal circuit on patients with hemorrhagic disease or tendency

ⓐ For priming

ⅰ) Add more than 1mL of 5% glucose fluid in 10mg vial and more than 5mL of 5% glucose fluid in 59mL vial until fully dissolved.

ⅱ) Add containing 29mg of nafamostat mesilate into 500mL of normal saline.

ⓑ Extracorporeal circulation

ⅰ) Add more than 1mL of 5% glucose fluid in 10mg vial and more than 5mL of 5% glucose fluid in 59mL vial until fully dissolved.

ⅱ) dilute mixed fluid with the 5% glucose fluid to fit the capacity of anticoagulant continuous infuser.

④ Caution during dissolution

: Do not apply normal saline or mineral salts into the vial directly due to crystal formation.

## 4.2 Production and packing of medication used in clinical study

The medication used in clinical study is produced by the study client and supplied to the pharmacist of the study institution.

The labeling of the medication followed the pharmaceutical article 71 section 5 and the contents are following.

1. Mark as “for clinical study”
2. Code name of medication
3. Production number and expiration date
4. Storage method
5. Company name of manufacturer or importer
6. Mark as “Use for clinical study only”

Clinical study labels should be attached at vial and outer packing.

## 4.3 Maintenance and revocation of double blind status

This study is open label clinical study, thus revocation is not required.

## 5. Target disease and clinical study period

### 5.1 Target disease

Patients in ICU who are in need of CRRT with hemorrhagic tendency which restrains use of heparin

### 5.2 Clinical study period

12 months after approval of clinical study

6. Inclusion/exclusion criteria, target number of subjects and its estimation basis

6.1 Inclusion criteria

Patients are selected as subjects with following conditions;

(1) Adult male/female patients aged from 18 to 80.
(2) ICU patients who are hemodynamically unstable to be in need of CRRT and has one of the hemorrhagic tendencies described as follows.
  ① Platelet < 100,000
  ② aPTT > 60 sec
  ③ PT-INR > 2.0
  ④ Active bleeding
  ⑤ Major surgery within 48 hours
  ⑥ History of cerebral hemorrhage or major bleeding within 3 months
  ⑦ Septic shock or DIC
(3) Patients or his/her legal guardian has voluntarily agreed to participate in the study and provided written informed consent.

6.2 Exclusion criteria

Patients with following conditions are excluded;

(1) Pregnancy, breast feeding or possibility of pregnancy
(2) History of allergy to nafamostat mesilate that might lead to shock.
(3) Any other conditions that made the candidate unfit according to the attending physician.

6.3 Target number of subjects and its estimation basis

6.3.1 Number of subjects

We are planning to enroll 33 subjects per group that is suitable for inclusion/exclusion criteria and analyze final group of 31 subjects.

| | Futhan group | No-anticoagulation group | Total subjects |
|---|---|---|---|
| Validity assessment subjects | 31 | 31 | 62 |
| Validity assessment subjects including drop-out (5%) | 33 | 33 | 66 |

6.3.2 Estimation basis

Primary end point of this clinical study is to evaluate the mortality of Futhan group and compare it to mortality of no-anticoagulation group.

Generally, the mortality of CRRT patients in ICU is known to be roughly 50% [1,4,5].

Thus, we anticipate the mortality in futhan group as 50% and assume the tolerance as 15% to estimate actual mortality. Also, we will add the same number of no-anticoagulation subjects to compare with futhan group on mortality.

Level of significance, $(\alpha = 0.1)$
Allowable limits of error for mortality are 15%. ($\delta$=0.15)
Considering the patient's severity in ICU, wastage rate is set at 5%. ($r$ = 0.05)

Hence, necessary number of subjects is calculated after the following formula;

$$n_t = \frac{Z^2_{1-\alpha/2}p(1-p)}{\delta^2}$$

By this formula, we estimate necessary number of subjects and calculate the final number of subjects by considering drop-out rate using the following formula.

$$n = \frac{n_t}{1-r}$$

The necessary number of subjects is 31 patients per group, and considering the 5% drop out rate, the final number would be 33 patients per group.

7. Clinical study method

7.1 Clinical study design

This study is single center, randomized, open clinical study to compare mortality, patients' recovery, safety using nafamostat mesilate and no-anticoagulation during CRRT in ICU patients who has hemorrhagic tendency that prohibits usage of heparin.

When ICU patients with hemorrhagic tendency that needs CRRT who fits inclusion/exclusion criteria voluntarily agrees to participate in the study, patients who fit the inclusion criteria by subject compatibility result are randomly assigned to each group. Since CRRT duration can be different due to patient's condition, nafamostat mesilate administration will be continued until CRRT is discontinued. Afterwards, mortality after 28 days of CRRT discontinuation and renal recovery at 1st outpatient department visit will be compared between each group.

7.2 Dosage, administration method and administration duration

7.2.1 Dosage

Initial dosage of nafamostat mesilate is 20mg per hour and will be adjusted between 10 to 30mg per hour upon the researcher's opinion.

7.2.2 Administration method

(1) Priming
Two vials of IV futhan is dissolved in 2mL of 5% glucose fluid and then added to 500mL of normal saline. This fluid was used to cleanse and charge the circuit.

(2) Extracorporeal circuit
One vial of IV futhan 50 was dissolved in 15mL of 5% glucose fluid. The mixture was continuously infused to the circuit in rate of 20mg per hour.

7.2.3 Administration duration

Futhan fluid was administered during CRRT duration

## 7.3 CRRT method

During CRRT, blood flow was maintained at the speed of 130 ~ 200mL/min, and was adjusted considering patient's condition.

## 7.4 Combination criteria

### 7.4.1 Possible combination medication

The medications listed as following are possible to be used in combination with nafamostat mesilate during clinical study.

① The medication that was administered to the patients before the clinical study to manage underlying disease and does not temper with the result of the clinical study will be allowed under the researcher's approval.
② The transient medication to treat other disease and adverse events will be allowed under the researcher's approval.

The information (medication name, purpose, dosage, duration etc.) of all combination medication (including medications to treat other disease and adverse events) will be thoroughly reported in the patient's case report.

### 7.4.2 Medication that are prohibited to use in combination

The medications listed as following are not allowed to use in combination with nafamostat mesilate.

① Medication in similar effect: warfarin, urokinase, FOY, aprotinine, dextrin, prostagalndine etc.

② Anticoagulation medication: heparin, citrate, hirudine etc.

When the patient is needed to use medication that are prohibited to use in combination with nafamostat mesilate under researcher's approval, the patient is excluded from the study immediately and an accurate and detailed documentation should be noted at the last page of the clinical report.

7.5 Randomization

In this study, patients are randomly assigned to either futhan group or no-anticoagulation group and are proceeded as parallel study. Necessary subject number is 33 patients in each group, considering the wastage rate of 5%.

At the enrollment, patients are assigned randomly with stratification of diabetes mellitus. Patients who enrolled in the study was assigned to futhan group or no-anticoagulation group according to the random assignment number by preformed random place card.

If group A is futhan group, group B is no-anticoagulation group, vice versa.

The following is the example when block size is 4 or 6

List number of cases: 1=AABB, 2=ABAB, 3=ABBA, 4=BAAB, 5=BABA, 6=BBAA, 7=AAABBB, 8=AABABB, ···, 26=BBBAAA (26 number of cases)
SAS Random Program value (randomly assign 1 to 26): 1, 26, 8, 2, 1 ····
Random assignment order: AABB BBBAAA A ABABB ABAB AABB ····

Random assign number of subjects was assigned to the patients who fit the inclusion criteria and not the exclusion criteria in order of registration by the random place card that was pre-distributed to the researchers

8. Observation, clinical examination and observation method

8.1 Clinical study schedule

| Period | Screening | Treatment[1] | Closing[2] | Follow-up[7] |
|---|---|---|---|---|
| Written consent | X | | | |
| Authorize a Screening number | X | | | |
| Demographical analysis | X | | | |
| Personal/Drug history survey | X | | | |
| Vital sign check[3] | X | X | X | |
| Physical examination | X | | X | |
| Pregnancy test[4] | X | | | |
| Clinicopathologic examination[5] | X | X[5] | X | |
| Severity analysis | X | | | |
| Filter life span | | X | X | |
| CVP monitoring | | X | | |
| Subject compatibility assessment[6] | | X | | |
| Randomization | | X | | |
| Drug administration | | X | | |
| Check difference in drug dosage | | X | X | |
| Adverse event check | | X | X | |
| Residual renal function check[8] | | | | X |
| Mortality check | | | X | X |
| Blood loss | | X | X | |
| Packed RBC transfusion | | X | X | |
| Free frozen plasma, Platelet[9] | | X | | |

1: administered until treatment is concluded

2: If patients are improved, closing examination was performed at discontinuation of CRRT. However, if the patient has deceased, the last examination before death was used.

3: Body temperature, pulse rate, respiratory rate, blood pressure was examined before other examination was performed. During CRRT, vital sign was examined every 4 hours. At screening, body temperature was examined rectally.

4: Pregnancy test was performed in female without sterilization or any effective contraception in child-bearing age.

5: Detailed examination was as following;
· Hematologic: Hemoglobin, Hematocrit, RBC, WBC, Platelet, differential count, ESR
· Biochemical: Alkaline phosphatase, BUN, Creatinine, SGPT (ALT), SGOT (AST), Total bilirubin, Uric acid, Na, K, Cl, Ca, P, CRP, total $CO_2$
· Arterial Blood Gas: $O_2$, $CO_2$, arterial pH, $HCO_3$, Base excess/deficit, Oxygen saturation
· Lipid: Cholesterol
· Urine analysis: pH, Protein, Glucose, Ketone, WBC, RBC
· Coagulation test: aPTT, PT-INR
· Specific coagulation test: fibrinogen, fibrin degradation product
During CRRT, Clinicopathologic examination was performed every 72 hours. If CRRT was discontinued before 72 hours, examination was performed at discontinuation. Uine analysis was performed at screening and closing. Specific coagulation examination was substituted for the examination performed recently
6: Subject compatibility assessment was performed based on all examination, personal history and inclusion/exclusion criteria. Patients who were fit in these criteria was enrolled as subjects.
7: The patients were followed until discharge for mortality to confirm survival until 28 days after initiation of CRRT.
8: After discontinuation of CRRT, patients were followed at discharge to confirm dialysis maintenance and at first outpatient department follow-up after discharge, renal functional restoration was checked.
9: Free frozen plasma, platelet transfusion was checked during CRRT.

## 8.2 Observation

### 8.2.1 Written consent and demographical survey

Before clinical study begins, the patients are thoroughly explained about the purpose and content of the clinical study and after patients sign the consent form, screening number is assigned and demographic survey is sought.

Record data is approval or disapproval, approval date, initial of subject, sex, birth date, age, etc.

### 8.2.2 Past history and drug history survey

Patient’s past medical history and medication administered to the patient within 4 weeks is thoroughly identified by examining patient’s medical examination and check medical history. The contents that need to be included are as following;

Past medical history including surgical history, allergy etc. for the past 2 years, duration of current diseases (year or year and month at which the disease has occurred; has to investigate thoroughly by interview), researcher's remark,

### 8.2.3 Vital sign

Vital sign includes body temperature, pulse rate, respiratory rate, and blood pressure. Vital sign is checked right before and initiation of CRRT and continued afterwards every 4hours. Body temperature was checked rectally at screening.

### 8.2.4 Physical examination

Physical examination is performed at screening and considered as baseline data, afterwards the final physical examination is performed at cessation of CRRT by identical researcher. Physical examination contains allergic, cardiovascular, pulmonary, gastrointestinal, hepatic, endocrinologic, renal, genitalial, musculoskeletal, skin, connective tissue, neurologic psychological and other examination.

Notable disturbance during physical examination at screening is recorded in physical examination section of data sheet. After initiation of CRRT, notable disturbance that can be identified as adverse event should be noted in the adverse event section of data sheet. However, if the abnormal physical examination was redundant as screening, it should be recorded in physical examination.

### 8.2.5 Pregnancy test and Clinicopathologic examination

Pregnancy is confirmed by urine HCG test in female of childbearing age without sterilization operation or menopause (including surgical menopause) at screening.

Clinicopathologic test is performed in all patients and evaluates patient’s general condition. All patients are required to perform pregnancy test, clinicopathologic test and confirm inclusion/exclusion criteria.

Clinicopathologic test is performed at screening and at the cessation of CRRT. When researcher considered it necessary, additional examination is possible.

Hematologic, biochemical, arterial blood gas, lipid, blood coagulation test is performed every 72 hours after initiation of CRRT. If CRRT is discontinued within 72 hours, examination is performed at the time of cessation. Urinary test is performed at screening and cessation. Specific coagulation test is performed at screening. When the patients deceased during CRRT and the examination at discontinuation were not performed, the examination right before the death can be used.

Examination includes as follows;

· Hematologic: Hemoglobin, Hematocrit, RBC, WBC, Platelet, differential count, ESR
· Biochemical: Alkaline phosphatase, BUN, Creatinine, SGPT (ALT), SGOT (AST), Total bilirubin, Uric acid, Na, K, Cl, Ca, P, CRP, total $CO_2$
· Arterial Blood Gas: $O_2$, $CO_2$, arterial pH, $HCO_3$, Base excess/deficit, Oxygen saturation
· Lipid: Cholesterol
· Urine analysis: pH, Protein, Glucose, Ketone, WBC, RBC
· Coagulation test: aPTT, PT-INR
· Specific coagulation test: fibrinogen, fibrin degradation product
· Pregnancy test: urine HCG test

8.2.6 Central venous pressure monitoring

Central venous pressure is monitored every 24hours during CRRT.

8.2.7 Severity evaluation

Patient’s disease severity is evaluated using Acute Physiology and Chronic Health Evaluation II (APACHE II) and severity of acute renal failure is categorized by Cleveland Clinical Foundation score (CCF score) and RIFLE criteria (enclosure 7,8,9). BUN/Cr values used to assess the CCF score and RIFLE criteria is done at screening.

8.2.8 Filter life span

To check filter life span, every filters used during CRRT was checked for initiation time, discontinuation time, and the reason for filter failure.

The reason for filter failure is categorized as follows;

① Due to filter clot
② Ultrafiltrate loss is less than 150mL/hr in recent 3 hours
③ Persistent transmembrane pressure > 200mmHg
④ Vascular access abnormality
⑤ Extracorporeal circuit abnormality
⑥ Due to another apparatus (such as radiologic examination)
⑦ Etc.

8.2.9 Subject suitability assessment

By physical history and basic investigation data, patients that are fit to section 6.1 inclusion criteria and section 6.2 exclusion criteria are screened after voluntarily agreed to consent. After screening, all results are put together to finally evaluate whether the patients are appropriately enrolled.

8.2.10 Randomization and prescription of study medication

Patients who are fit to inclusion/exclusion criteria are randomly assigned to futhan and no-anticoagulation group as described by section 7.5 and study medication was administered. Randomization is performed after the subject suitability assessment was conducted and when the initial administration of nafamostat mesilate is done, it is continued until discontinuation of CRRT.

8.2.11 Identification of change in medication history

When there is alteration in medication compared to the medication at screening, it is investigated and documented in the case report.

8.2.12 Collection of medication and checking compliance

The rest of the medication that remains after administration is recorded.

8.2.13 Checking adverse events

Patients are repeatedly educated to report any information of adverse events, and researchers are obligated to investigate adverse events by interview and physical examination. When investigating adverse events, date of manifestation, disappearance, severity, actions that took step related to the study medication, relation with the study medication, any medication that is suspected to have relations with the adverse events except the study medication and treatment related to adverse event was sought.

The typical symptoms that are classified as adverse events are described as follows.

(1) Fever
Fever is checked by documenting body temperature, its severity is investigated, and finally the relationship between the medication and symptom is evaluated.

(2) Subjective and objective symptom
Subjective and objective symptom is investigated through the researcher's examination, its severity is investigated, and finally the relationship between the medication and symptom is evaluated.

(3) Clinicopathologic examination
Any abnormality of clinicopathologic examination is followed until it is recovered, and the predicted cause is evaluated.

8.2.14 Checking renal function

After discontinuation of CRRT, patients were sought whether he or she continued hemodialysis and BUN/Creatinine is checked to evaluate renal recovery at the first outpatient department visit after discharge.

8.2.15 Checking Mortality

After discontinuation of CRRT, patients were sought whether he or she died and mortality at 28 days after initiation of CRRT was checked.

#### 8.2.16 Blood loss

After initiation of CRRT, amount of blood loss at filter exchange was examined.

#### 8.2.17 Transfusion

After initiation of CRRT, transfusion is performed when hemoglobin is under 7g/dL or under 10g/dL and shows hemorrhage. Also, free frozen plasma or platelet concentrate is provided whenever is necessary.

## 8.3 Observation method (per visit)

### 8.3.1 Screening

Patients who are selected for this study is explained about the study and evaluate as following order

① Before enrollment, patient is explained of the study procedure, and signs the written consent.
② Patients are given a screening number in order.
③ Patient’s demographic information and medical history (past and current medical history including surgical history) are sought.
④ Vital signs are checked
⑤ Physical examinations are performed.
⑥ Clinicopathologic examinations are performed.
⑦ Pregnancy test is performed, if the patients is female of childbearing age.
⑧ Patient’s severity was evaluated using CCF score, RIFLE criteria, APACHE II.

### 8.3.2 Medication phase (randomization and after initiation of CRRT)

① After screening, change in medication history is checked.
② Every examination including clinicopathologic test and assessment is collected to finally evaluate the patient’s suitability as a subject.
③ Patent is given a randomization number by random enrollment.
④ Study medication is administered.
⑤ Hematologic examination was conducted every 72 hours after initiation of CRRT.
⑥ Vital sign (blood pressure, body temperature, pulse rate, respiratory rate) is assessed every 4 hours after initiation of CRRT.
⑦ Filter life span and reason for discontinuation is sought.
⑧ Blood loss is checked at filter change after initiation of CRRT.
⑨ Transfusion (packed RBC, free frozen plasma, platelet concentrate) is assessed after initiation of CRRT.
⑩ Any changes in medication are recorded.
⑪ Adverse event is investigated.
⑫ Bleeding accident is checked.

#### 8.3.3 Study completion or discontinuation

The following examination is conducted when the subject has completed or discontinued the study. When the study was discontinued due to subject’s death, examination before death is used.

① Vital sign is checked.
② Physical examination is conducted
③ Clinicopathologic test (hematologic, hematochemical, lipid, blood coagulation, blood gas, urine test) is conducted.
④ Any difference in medication is assessed.
⑤ Adverse event is investigated.
⑥ Remaining medication is collected, checked and documented.

#### 8.3.4 Follow-up

Follow-up investigation includes recovery of renal function, patients’ mortality. When patient’s condition is improved and CRRT is not necessary, patient is followed whether he or she is on dialysis at discharge, and renal recovery is sought at 1st outpatient department visit after discharge. Also, mortality at discharge and 28 days after initiation of CRRT is investigated. Follow-up investigation is conducted according to patient’s outpatient department visit schedule and no extra-visit for the study is planned.

## 9. Precautions using study medication

### 9.1 Precaution considering the study medication: SK chemicals corp. 'IV futhan'

1. Warning

The patient should be inspected thoroughly to reveal any history of hypersensitivity, because of the concerns for shock or anaphylactoid reaction. Also, in case of a shock, prepare for emergency treatment, observe, and if these symptoms occur, immediately stop administering the medication and treat properly.

2. Prohibited in following patients

The patients who have history of hypersensitivity by the medication

3. Adverse events

(1) Improvement of acute symptom in pancreatitis
117 (1.74%) out of 6,732 cases showed adverse events. The symptoms included 55 cases (0.82%) of liver function abnormalities including GOT/GPT elevation, 23 cases (0.34%) of hypersensitivity reactions such as rash, urticaria, and 14 cases (0.31%) of electrolyte imbalance such as hyperkalemia. (Re-examination conclusion report result in Japan)

(2) DIC
241 (6.69%) out of 3,602 cases showed adverse events. The major symptoms included 185 cases (5.14%) of electrolyte imbalance such as hyperkalemia and hyponatremia, 53 cases (1.47%) of liver function abnormalities, and 11cases (0.31%) of hypersensitivity reaction. (Re-examination conclusion report result in Japan)

(3) Anticoagulation of extracorporeal circuit in patients with hemorrhagic tendency
48 (1.18%) out of 4,053 cases showed adverse events. The major symptoms included 41 cases (1.01%) of gastrointestinal symptom, and 9 cases (0.22%) of hypersensitivity reaction. (Re-examination conclusion report result in Japan)

(4) Severe adverse events

① Shock, anaphylactoid reaction
Detailed observation is required due to possibility of shock and anaphylactoid reaction (respiratory distress, asthma-like reaction). And if these symptoms appear, immediate discontinuation of medication with proper management is required.

② Hyperkalemia
(Pancreatitis: 0.19%, DIC: 4.53%, anticoagulation for extracorporeal circuit: 0.02%; Re-examination conclusion report result in Japan)
Extra caution is required due to possibility of hyperkalemia, when using potassium sparing diuretics, potassium containing medication. When it occurs, immediate

discontinuation of the medication and proper management should follow. Also, there have been some cases of arrhythmia due to hyperkalemia.

(5) Other adverse events

① Skin: Occasionally rash, rarely erythema, urticaria occurred, and the medication is discontinued after the symptom appears.

② Musculoskeletal: Myalgia and arthralgia have been reported.

③ Gastrointestinal: Anorexia, diarrhea, nausea, vomiting have been reported.

④ Hepatobiliary: Occasionally GOT/GPT elevation, ALP elevation, LDH elevation, total bilirubin elevation and Jaundice have occurred.

⑤ Hematologic: Occasionally leucopenia, eosinophilia, thrombocytopenia, and thrombocytosis have occurred. Bleeding tendency can occur, and in this occasion, medication dose will be tapered or discontinued.

⑥ Cardiovascular: Palpitation, hypertension and hypotension can occurred.

⑦ Urologic: Intermittently BUN/Creatinine elevation occurred.

⑧ Systemic: Headache, chest pain, general weakness, chest discomfort, and rarely fever have been reported.

⑨ Administration site: Rarely pain and angiitis with swelling occurred.

4. General precaution

(1) Hyperkalemia and hyponatremia due to potassium excretion inhibition and sodium excretion acceleration can be occurred. Hence serum potassium and sodium level should be checked regularly and if any abnormalities have been observed, immediate discontinuation and proper management should be performed.

(2) When using Potassium containing medication and potassium sparing diuretics in combination, be cautious about hyperkalemia. Also, when abnormal serum potassium level is confirmed, electrocardiography should be checked to identify arrhythmia. When used in extracorporeal circuit, this medication can aggravate hemorrhage. Hence close observation is required, and if needed, the medication should be tapered or discontinued

5. Administration to elderly

Elderly are generally with reduced physiologic function and careful caution for dose reduction is required.

6. Administration to pregnant or lactating female

(1) Since safety in administration during pregnancy has not been confirmed, medication to pregnant female or female who is possible to have pregnant should be considered when

the benefit of the treatment exceeds the risk. In animal study, there has been reports of increase in fetal mortality (rat, rabbit), inhibition in fetal weight gain (rat), and decrease in delivery rate (rat)

(2) In animal study (rat), there has been report of transition of metabolites via breast milk, thus when drug is administered, breast feeding should be stopped.

7. Administration to child

(1) Precaution during prescription

① The medication should be used after completely dissolved by 5% glucose fluid.
② When dissolving the medication, needle should be inserted in the middle of the rubber of the vial. Also, if 19 gauge or thicker needle is used, the rubber topping or part of it can drop into the vial. Hence, precaution is required.
③ Normal saline or fluid containing mineral should not be directly injected to the vial due to the risk for crystal formation.

(2) Precaution after prescription

The medication is administered as soon as possible.

(3) Precaution during injection

① Dosage: When the medication is administered to extracorporeal circuit, appropriate dosage is required with consideration to bleeding tendency, residual blood in extracorporeal circuit, and thrombokinesis or blood coagulation time.
② Infusion rate: The medication should not be administered into vein or extracorporeal circuit rapidly.
③ Dialyzer: The medication is highly absorbable to AN69 (polyacrylonitrile) membrane, hence it is prohibited to be used with the membrane.
④ Injection: Extravasation of the medication can lead to inflammation and necrosis of the injection site, hence it is crucial not to extravasate the medication.

10. Study discontinuation, drop out, or analysis exclusion criteria

10.1 Study discontinuation and drop out criteria

All patients who enrolled in the study should be recorded of the completion of the study, and if the medication is discontinued or observation is stopped, the reason is recorded. The criteria for patient’s drop-out are as follows.

① Circumstance that violates inclusion/exclusion criteria
② Serious adverse events occurred in patients or patients demands to be dropped out of the study due to adverse events.
③ Patient or legal guardian demand the discontinuation of the study due to unsatisfactory curative value during the study.
④ Severe hemorrhage
⑤ Critical violation of study protocol by principal investigator, researcher or patients.
⑥ Patients or legal guardian’s withdrawal of the consent.
⑦ When patients cannot be followed.
⑧ When the patient takes a medication that can influence the study result, without the researcher’s authorization during study period.
⑨ When the patient is decided not fit for the study progression by researchers.

10.2 Disposal of violation on protocol and non-compliance

The principal investigator and researchers should fully recognize and perform the study protocol, hence the protocol is not violated. However, the inevitable violation will be dealt with as follows.

In case of severe protocol violation, researcher should disregard the patient’s data from the analysis and the criteria are as follows.

① Failure to acquire written consent
② Violation of inclusion/exclusion criteria
③ Administration of the medication that is prohibited to use in combination during the study.

Other violation of protocol that is decided to be minor and is not thought to influence the study analysis is thoroughly documented with the degree of violation, reason. Afterwards, the violation is evaluated by researcher, funder, monitor, statistician whether the violation has tempered with the study and included in the PP analysis.

11.Efficacy assessment criteria, assessment method, and analysis method (Data analysis method)

11.1 Assessment criteria

Primary and secondary validity assessment variable is as follows and each assessment variables are analyzed with classification of futhan and no-anticoagulation group.

11.1.1 Primary validity assessment variable

Mortality at ICU

11.1.2 Secondary validity assessment variables

① Mortality in hospital
② Mortality at 28 days after initiation of CRRT
③ CRRT duration
④ Hospitalization period in ICU
⑤ Hospitalization period
⑥.Survival period
⑦ Filter life span
⑧ Blood loss
⑨ Transfusion

11.2 Assessment method

11.2.1 Primary validity assessment variable

Mortality at ICU:

We will check whether patients' mortality at ICU after CRRT is 50% and additionally whether there is any statistically significant difference between two groups.

11.2.2 Secondary validity assessment variables

(1) Mortality in hospital

We will check whether the patients' mortality in hospital after CRRT is 50%, and additionally whether there is any statistical significant difference between two groups.

(2) Mortality at 28 days after initiation of CRRT

We will check whether the patients' mortality at 28 days after initiation of CRRT is 50%, and additionally whether there is any statistical significant difference between two groups.

(3) CRRT duration

We will check the duration of CRRT at ICU between the two groups.

(4) Hospitalization period in ICU

We will check the patients' duration within ICU until recovery to general ward or death.

(5) Hospitalization period

We will check the patients' duration within hospital until discharge or death.

(6) Survival period

We will calculate the survival 28 days after CRRT in both groups. Each patient's survival period is sought to calculate median survival time.

(7) Filter life span

We will check each filter life span during CRRT when CRRT is discontinued and calculate average filter life span.

(8) Blood loss

We will check blood loss at filter change during CRRT.

(9) Transfusion

We will check the amount of transfusion during CRRT.

## 11.3 Analysis method

### 11.3.1 General principal of result analysis

The data retrieved from the patients during the study is analyzed by Safety analysis, ITT (Intent-To-Treat) analysis and PP (Per Protocol) analysis.

The Safety analysis is analysis including all data from patients in futhan group who have taken the medication at least once and no-anticoagulation group

The ITT analysis is analysis including all data from patients who have been confirmed whether he or she is dead or alive in futhan group who have taken the medication at least once and no-anticoagulation group. And when there is a missing value or patient drop out before the study is over, the latest value can be analyzed as data acquired at relevant time (Last Observation Carried Forward Method).

The PP analysis is analysis including the data from patients who have successfully concluded the study protocol.

The data regarding validity is principally analyzed by PP analysis, and additionally analyzed by ITT analysis.

The data regarding safety is principally analyzed by Safety analysis.

### 11.3.2 Demographic baseline data

The mean, standard deviation, maximum and minimum values are calculated with continuous data regarding demographic and general condition.

### 11.3.3 Validity data

#### 11.3.3.1 Primary validity assessment value

Mortality at ICU is presented.

#### 11.3.3.2 Secondary validity assessment value

(1) Mortality within hospital
Mortality within hospital is presented.

(2) Mortality at 28 days after initiation of CRRT
Mortality at 28 days after initiation of CRRT is presented.

(3) CRRT duration
The mean, standard deviation, maximum, minimum values of CRRT duration in each group are presented.

(4) ICU hospitalization period
The mean, standard deviation, maximum, minimum values of ICU hospitalization period after ICU initiation in each group are presented.

(5) Hospitalization period
The mean, standard deviation, maximum, minimum values of hospitalization period after CRRT initiation in each group are presented.

(6) Survival
Survival rate at 28 days after initiation of CRRT is calculated using Kaplan-Meier analysis with data of all subjects in the clinical study including censored data. Also, Kaplan-Meier curve is presented as graph, and median survival time of each group is presented.

(7) Filter life span
The mean, standard deviation, maximum, minimum values of filter life span which is changed during the study in each patient are presented.

(8) Blood loss
The mean, standard deviation, maximum, minimum blood loss during each filter change in CRRT with each patient are presented.

(9) Transfusion
The mean, standard deviation, maximum, minimum values of transfusion during CRRT in each patient are presented.

11.3.4 Safety data

11.3.4.1 Adverse event

All adverse events that have been reported during the study will be charted, and incidence of adverse event will be calculated.

11.3.4.2 Clinicopathologic examination result

All laboratory result is presented and compared with the result from screening.

11.3.4.3 Bleeding accident

Bleeding accidents including upper gastrointestinal, cerebral, peripheral bleeding during CRRT is observed and documented.

## 12. Safety evaluation method including adverse event, evaluation criteria and reporting method

### 12.1 Definition of safety related terms

(1) Adverse events (AE)

Adverse event is a sign, symptom, or disease which occurs after attending the clinical study and is neither intentional nor desirable. Adverse event does not necessarily have to have cause-and-effect relationship with the study medication.

(2) Adverse drug reaction (ADR)

"Adverse drug reaction" is a reaction that has reasonable doubt that the medication and the reaction that has cause-and-effect relationship, or at least the possibility is not ruled out.

(3) Serious and Unexpected Adverse Drug Reaction

"Serious and Unexpected Adverse Drug Reaction" is the nature and severity of reaction that is not anticipated according to available medication related information and if this happens, spontaneous report should be followed.

(4) Serious AE/ADR

Serious AE/ADR is adverse event or adverse drug reaction that has occurred in any dosage of medication during the clinical study, and is as follows;

① Patient's death during the study
② Life threatening circumstances (when the patient is exposed to the risk of death by the incidence of reaction)
③ When persistent or significant disability or functional deterioration is anticipated.
④ When the patient is need to hospitalized or prolong hospitalization period in already hospitalized patient
⑤ When causes congenital disability or abnormality
⑥ Other medically significant circumstances

Even if it is not listed above, when patient's safety or health is thought to be compromised, under supervision of attending physician and related specialist's opinion, the reaction is considered as serious adverse event and appropriate management is followed accordingly.

## 12.2 Evaluation method

- Adverse event is newly developed symptom that was not observed before study enrollment, and is not intended symptom and sign that is transient related to the usage of the study medication, regardless of cause-and-effect relationship with the study medication.

- The undesirable clinical sign and symptom that was observed during or before screening is documented in physical examination data sheet, and is not documented as adverse events.

- The adverse event is thoroughly reported in the adverse event data sheet (symptom and sign, observation date, duration), and event that is not documented in the data sheet is considered as subjective symptom.

- The severity of adverse event is referred to evaluation criteria by the researcher and the severity is graded.

- The cause-and-effect relationship between the symptom and medication is referred to evaluation criteria and graded in 6 phases.

## 12.3 Evaluation criteria

### 12.3.1 Fever

Fever is evaluated through body temperature, and is graded as following;

| Criteria | Grade 0 | Grade 1 | Grade 2 | Grade 3 | Grade 4 |
|---|---|---|---|---|---|
| Fever (℃) | none | <38.7 | 38.7~40.0 | >40.0 | Fever with hypotension |

12.3.2 Subjective/objective sign

The severity of subjective/objective sign is evaluated in grade by pre-existing criteria (for example, WHO adverse event evaluation criteria) or by general evaluation criteria during researcher's physical examination.

| General evaluation criteria | None (0) | Mild (1) | Moderate (2) | Severe (3) | |
|---|---|---|---|---|---|
| WHO | Grade 0 | Grade 1 | Grade 2 | Grade 3 | Grade 4 |

· WHO 5 phase classification

Grade 0: Condition available of normal activity without any restraint
Grade 1: Condition possible of ambulation and work, but restrained from physically challenging activity.
Grade 2: Condition possible of ambulation and self-protection, but not possible to perform more than 50% of working hours
Grade 3: Condition possible of limited self-protection, and restricted to bed or chair for more than 50% of working hours
Grade 4: Condition impossible of self-protection, and completely restricted to bed or chair

· General criteria classification

Mild (1): Adverse event that is easily bearable.
Moderate (2): Adverse event that restrict daily life considerably
Severe (3): Adverse event that restricts normal daily life.

12.4 Cause-and-effect relationship with the study medication

When adverse event occurs, the researcher should classify the event as follows, and if necessary, the opinion of principal investigator is added.

① Definitely related
- The evidence over administration of medication in question is present.
- The medication in question is the most compelling reason of adverse event compared to other possible reason.

- The adverse event is resolved with discontinuation of the medication in question.
- The adverse event is reproduced after rechallenge test (done when it is possible).
- The adverse event is consistent with the information known about the medication in question or medication of same sorts.

② Probably related
- The evidence over administration of medication in question is present.
- The time sequence between the administration of the medication and adverse event is reasonable.
- The medication in question is the most compelling reason of adverse event compared to other possible reason.
- The adverse event is resolved with discontinuation of the medication in question.

③ Possibly related
- The evidence over administration of medication in question is present.
- The time sequence between the administration of the medication and adverse event is reasonable.
- The medication in question is similarly compelling reason of adverse event compared to other possible reason.
- The adverse event is resolved with discontinuation of the medication in question.

④ Probably not related
- The evidence over administration of medication in question is present.
- There is other reason that explains the adverse event which is more compelling.
- The adverse event is not resolved or ambiguously resolved after discontinuation of the medication in question.

⑤ Definitely not related
- There is no evidence that the medication in question is administered.
- There is other reason for the adverse event that is most compelling.
- The adverse event is not resolved with discontinuation of the medication in question.

⑥ Unknown

## 12.5 Reporting method

### 12.5.1 Reporting the adverse event

· Principal investigator should inform the researcher, patients or legal guardian of possible adverse events and education the researchers to document all events that occur after administration of study medication.

· The systemic or clinicopathologic signs after administration of study medication is documented and stored to data sheets according to clinical study management regulation including the characteristics, event time, severity, management, treatment medication, duration, cause and effect relationship between the study medication.

· Principal investigator should describe and evaluate all sign/symptoms during clinical study when reporting study result. When serious adverse drug reaction occurs during the clinical study, principal investigator should report to IRB and decide continuance or discontinuation of the study. When prompt report is necessary due to following incidence, the funder should promptly report to the incidence to Korean food and drugs administration.

· "Serious adverse drug reaction" is a sign that cannot be excluded as a result of study medication administration and include following conditions.

① Patient's death during the study
② When the patient's life is threatened
③ When lasting or significant disability or functional deterioration occurs
④ When the patient needs hospitalization or delay in discharge when already hospitalized.
⑤ When the medication causes congenital deformity or abnormality
⑥ Other medically significant circumstances

· Additional safety information should be periodically reported until the adverse event is resolved (when the adverse event is resolved or impossible to follow up).

· Principal investigator should follow declaration of Helsinki regarding all matters.

12.5.2 Prompt report

(1) Purpose

The purpose of prompt report is to provide a new and important information of serious adverse drug reaction to Korean food and drugs administration, researchers and other related personnel.

⑵ Report subjects

① A case of serious and unexpected adverse event
② When a prompt communication with the Korean food and drugs administration is required

Appropriate medical and scientific decision is needed in each circumstances. Generally, the subjects include information that can influence the risk and benefit evaluation of the medication, that can influence in change of administering the medication, or following conditions.

a. Increase in incidence of clinical significance which was anticipated
b. A possibility of serious danger to the patients, such as insufficient dosage in medication treating life-threatening disease
c. Important safety information is acquired during newly performed animal study (for example, carcinogenicity)

⑶ Report timing

When serious and unanticipated adverse drug reaction occurred during the clinical study, principal investigator is obligated to report the matter to IRB to evaluate continuance or discontinuation of the study, and the client is obligated to report the matter to Korean food and drugs administration within given period in each section.

① Adverse drug reaction which is not anticipated and cause death or threatens life is needed to promptly be reported. The client is obligated to report to Korean food and drugs administration as soon as possible by phone, fax or document within 7 days. Full written document is needed to be submitted within 15 days after the client was informed.
② The client is obligated to report the adverse reaction within 15 days after the reaction was informed for other significant unanticipated adverse drug reaction

⑷ Minimum criteria for initial report

Information about final description and evaluation for initial report of adverse event may not be acquired until given period. But, initial report needs to be documented with following category until given period.

① The patient who suffered the adverse drug reaction
② Suspected medication
③ The informant
④ Significant and unanticipated adverse drug reaction

Follow up report can be submitted with more thorough investigation afterwards.

⑸ Report method

When prompt report is submitted over an adverse drug reaction, serious adverse reaction report form of Korean food and drug administration is used and all efforts should be made to report as many information as possible.

12.6 Measures taken when serious adverse drug reaction occurs

During the clinical study, principal investigator and researcher should make sure that the patient is safe. And when serious adverse event occurs, prompt discontinuation of the study, appropriate management is required to minimize the adverse event.

⑴ Duty of principal investigator

Principal investigator is required to inform IRB and the client when serious adverse drug reaction occurs, and discontinue part of or whole clinical study until further notice.

⑵ Duty of researchers

Researchers are required to inform the principal investigator and the client as soon as a serious adverse drug reaction occurs during clinical study.

⑶ Duty of IRB

IRB is required to order necessary measures including discontinuation of the study partially or as a whole, when serious adverse drug reaction occurs

.

(4) Duty of client

When the client was informed of serious and unanticipated adverse drug reaction from principal investigator or researchers, he or she should submit an adverse drug reaction report including a copy of reports from principal investigator or researchers to Korean food and drugs administration as soon as possible. And if the study was performed in multiple centers, each center should be notified.

## 13. Consent form, compensation agreement, and patient’s treatment after clinical study

### 13.1 Consent form

Principal investigator is required to take a written consent from the subjects after thorough notification of the clinical study method, study content, the effect of study medication, adverse events. Researcher is required to store the original consent form as the study record, and a copy of written consent and description of the study should be provided to the subject or his/her legal guardian.

### 13.2 Agreement on compensation

Refer to enclosure 3. Agreement on injured party

### 13.3 Criteria for patient’s treatment after clinical study

Subject who dropped out or unresponsive to the study medication is supervised to be treated with other appropriate management, and due to possibility of delayed adverse event, patient is always allowed to access medical assistance under researcher’s supervision.

## 14. Measures on patient's safety

### 14.1 Clinical study institution

The head of clinical study institution is required to equip with clinical laboratory, facilities and professional manpower that is needed for the clinical study and should be prepared to help perform the study appropriately.

### 14.2 Approval and modification of the study protocol

When it is necessary to gain approval of clinical study or modify already approved clinical study, original protocol or modified protocol is approved by IRB according to the phase of clinical study, and if needed, approved by Korean food and drugs administration. It is forbidden to enroll patients before approval.

### 14.3 Thorough awareness of the study protocol

This study is prepared in regard to right and welfare of the patient according to declaration of Helsinki. And, the principal investigator and researchers are required to be well-acquainted with the study protocol and respond to patient's problem aggressively.

### 14.4 Consent to clinical study

Before enrollment, the patient is thoroughly explained about the study content, effect of study medication, adverse reaction and safety, after which he or she voluntarily agrees to enroll in the study and provide written consent.

### 14.5 Accurate selection of subjects

Before the clinical study, the subject is fully evaluated whether he or she is fit for the study by sufficient interview and examination.

### 14.6 Inspection of clinical study progression

Principal investigator is required to report to the client periodically regarding adverse events, study progression, circumstances, results and the client is required to periodically inspect the progression of clinical study.

### 14.7 Monitoring of clinical study institution

Monitoring is performed to ensure that the study follows clinical study management standard and regulation article 28, that the subject's right and well-being is protected, reported clinical study data is accurate, and coincide with data sheets, and that study protocol is approved.

Monitoring of clinical study is performed through regular visit and phone call to institution by Dream CIS, to which SK chemical. Corp has trusted monitoring. Monitoring during the visit is comprised of assuring the original copy of data sheet, drug management record, data storage. Also, clinical study progression is observed and if there is a problem, it is consulted with the researchers.

Appropriate time of visit is consulted between the researcher and the monitor. Researchers are required to give the monitor access to source documents of the subjects that can identify the data documented in the data sheet.

### 14.8 Subject's confidentiality

The record that can identify the subject is confidential and after the study result is published, the patient's identity is kept secret. Detailed content is as follows.

Client, monitor, and inspector related to the clinical study can access patient's record for monitoring, inspection, and supervision of progression. Researchers should be well informed that monitor and inspector from the client and clinical study consignment agency can review and copy subjects chart and case report under agreement of clinical study contract. These data is required to be classified and facilities and management standards for the storage of these data is required.

Also, patients are documented and classified by subject identification code (generally the initial of the subjects) in all documents related to clinical study including case report.

## 14.9 Management of clinical study medication

- Clinical study medication is stored in room temperature (1~30℃), and the medication is not used without principal investigator or researcher's prescription.
- The client should discuss the study medication protocol with the principal investigator, and issue the medication directly to supervising pharmacist and preserve the receipt. The medication needs to be marked as "for clinical study only".
- The supervising pharmacist is required to store and manage the study medication so that the medication is not used other than the clinical study.
- The client should identify the quantity of the clinical study medication and its storage condition, and take necessary measures so that the clinical study can be performed appropriately.
- When the clinical study is discontinued, completed or the researcher is not performing the study as protocol, unused remaining study medication is collected and discarded. The supervising pharmacist is required to return the remaining study medication to the client and store the receipt.

## 14.10 Management after adverse event

When adverse event occurs, attending doctor is required to execute necessary examination and treatment immediately. When serious adverse event occurs, the study is discontinued and necessary measures are executed immediately and appropriately under advisement of section 12.6.

15. Other provision that is necessary to perform the clinical study safely and scientifically

A storage system to preserve the clinical study data sheet and record is needed to be ready separately and secured. After the result report is completed, archive manager is pointed and related data is needed to be stored for 10 years after completion of clinical study.

16. Reference


1. Geun Ho Park JHS, Hyeon Gyu Yi, Sun Young Lee, Seoung Woo Lee, Moon Jae Kim (2004) prognostic analysis of patients with acute renal failure in intensive care unit by dialytic method. Kidney Research and Clinical Practice 23: 927-933.
2. Hyun Jin Kim YHL, Min Ok Kim, Hyun Jeong Beak, Yeon Sil Do, Eun Hee Jang, So Yeon Choi, Ho Myoung Yeo, Jung Ah Kim, Beom Kim, Bang Hoon Lee, Woo-Heon Kang, Dong Jin Oh, Wooseong Huh, Dae Joong Kim, Ha-Young Oh, Yoon-Goo Kim (2005) Comparison of Safety and Efficiency of Hemodialysis Using Heparin-bound Hemophan with those of Saline Flushing Hemodialysis and Hemodialysis Using Low Dose Heparin in Patients at Risk of Bleeding. Kidney Research and Clinical Practice 24: 246-254.
3. Jin Ho Kwak GBJ, Jeong Hun Seong, Eun Yi Hwang, Seung Yup Han, Seong Bae Park, Hyu Chul Kim (2006) Effect and safety of Futhan in hemodialysis patients with hemorrhagic risk. Kidney Research and Clinical Practice 25: 127.
4. Mehta RL, McDonald B, Gabbai FB, Pahl M, Pascual MT, et al. (2001) A randomized clinical trial of continuous versus intermittent dialysis for acute renal failure. Kidney international 60: 1154-1163.
5. Scheinkestel CD, Kar L, Marshall K, Bailey M, Davies A, et al. (2003) Prospective randomized trial to assess caloric and protein needs of critically Ill, anuric, ventilated patients requiring continuous renal replacement therapy. Nutrition 19: 909-916.
